# Reviews of Books

**Published:** 1924-12

**Authors:** 


### REVIEWS OF BOOKS.

### THE PLAGUE OF LONDON.

**The Great Plague of London in 1665.** By WALTER G. BELL, F.S.A. Illustrated. (The Bodley Head. 25s. net.)

The Plague of 1665 has long occupied a place in the popular imagination which is not altogether warranted by the actual facts of the epidemic. There had been, notably in 1603 and 1625, previous epidemics in England with a virulence and mortality quite in proportion to those of the final outburst which has acquired the title of " The Great Plague." The reason of this undue pre-eminence may perhaps be found in the fact that the Plague and the Great Fire which succeeded it form, as it were, a climax to one of the most interesting and romantic periods of our history. The storm and stress of the Civil War had drawn men of all social grades into its vortex, and had doubtless stimulated mental activity and imagination and sown the seeds which were to produce in the following century that greater sense of mutual dependence and corporate responsibility which is the basis of national sentiment. Doubtless the " Journal of the Plague Year " by Defoe has played its part.

Yet, in spite of this hold on popular thought, it is not a little surprising that the available material for the history of the Great Plague has received little detailed study. Mr. Walter Bell, whose work on the Great Fire was published a few years ago, has now produced in a companion volume a carefully documented account of the progress of the epidemic. It must be remembered that the plague was no strange newcomer which swept down upon an unprepared population, but a well-known domestic foe which lurked in the courts and alleys of the City and other towns and claimed its victims year after year, every now and again bursting forth in epidemic form of greater magnitude. It is this familiarity with the habitués of the disease, combined with the almost complete absence of a sense of corporate responsibility, rather than a mere selfish act of self-preservation, which in large measure explains the desertion of their posts by so many clergy and medical practitioners, including even Dr. Thomas Sydenham himself. Self-indulgent and licentious as the Court undoubtedly was, King Charles was only following regal precedent in removing his sacred presence to a place of safety at such a time.

There were, however, noble examples of devotion to duty. The Duke of Albemarle, whose fine portrait by Sir Peter Lely forms the frontispiece to Mr. Bell's volume, the Lord Mayor and a number of clergy and doctors stayed behind to do what they could to alleviate the distress, caused in large measure by the panic-flight of so many citizens to the country, and by the resulting cessation of trade and employment. The author computes that of a population of less than half a million about 140,000 left London, and that of those who remained 110,000 perished. This figure is undoubtedly much higher than that given by other writers and is greatly in excess of the total number of deaths attributed to plague in the Bills of Mortality, but Mr. Bell gives good reasons for his calculations. These weekly Bills were notoriously misleading, at first because there were manifest reasons for minimising the plague incidence, and later because the mortality was so heavy that the machinery of notification broke down completely.

The condition of the sufferers was pitiable indeed. Sick and sound closely confined in the old and ill-ventilated houses in a rainless summer of unusual heat, tended chiefly by ignorant and generally degraded and vicious women, there is little wonder that, when once infection entered a house, few of the inmates escaped. The plight of those admitted to the totally inadequate and overcrowded " Pest-houses " was equally horrible, and the number of the dead to be disposed of daily soon exceeded all the available means for decent burial. Mr. Bell has told the story of those tragic months, culminating in a total of 8,297 deaths in the third week of September, with a vivid pen. He has marshalled his mass of material clearly. The book is a valuable study of one of the many tragedies of English history, and has the additional advantage of a number of interesting illustrations and a copious index.

### NEW YORK HOSPITALS.

**The Hospital Situation in Greater New York.** Report of a Survey of Hospitals in New York City by the Public Health Committee of the New York Academy of Medicine. (Putnam. 25s. net.)

The interest and value of this report to the British reader lie not in the fact that it is what it purports to be, a survey of the hospitals in New York, though that would invest it with both those qualities for many, but that, by reason of the breadth of view with which the topics dealt with are treated, it is very much more. It might well be described as a systematic and comprehensive general study of important hospital questions. The range of subjects of which it treats is so wide, the manner in which each in turn is approached, investigated, and discussed is so admirable in its soundness, orderliness and thoroughness; in short, the whole book, alike in matter and method, is so excellent and of so wide an application that we strongly recommend it. It is worthy not only of perusal but of study by everyone who takes an interest in hospital questions. Certainly no hospital administrator, be he committee-man or official, should fail to give it thoughtful study.

It begins, or rather would begin if the reader were to take our advice and read chapter 3 first, with a consideration of the problem of illness among the people, the extent and character of disease, the determining of the illness rate, and the question of the place of the hospital in the treatment of disease. This, in particular, has an application to all places, and is not pertinent merely to New York or even the United States. This chapter is, perhaps, to the British reader, the most interesting of any in the book, inasmuch as it demonstrates the practicability of determining on substantial grounds the relation which the numerical strength of hospital beds should bear to population. Other chapters treat of revenue and expenditure, concluding with a consideration of surpluses and deficits; administration; medical organisation; nursing, including the scarcity of nurses, which prevails in New York as in London—an excellent chapter; hospital records; convalescents; special problems; and community problems, embracing the mutual responsibility of the community to hospitals and hospitals to the community.

It is impossible to comment in detail on the contents of this most valuable treatise—a considerable volume might profitably be devoted to such a purpose. It will, however, perhaps surprise many to learn that, whereas in London, with its population of 7,250 000, the hospitals included in the Statistical Report of King Edward's Fund number 116, contain 12,660 beds, and cost, in round figures, to maintain, £2,630,000, the hospitals of Greater New York, with a population of 5,620,000, number 182, contain 32,000 beds, and cost to maintain $35,000,000—say £7,000,000 at par of exchange. The Public Health Committee of the New York Academy of Medicine who publish this survey consists of 33 doctors whose time it occupied for the greater part of a year, and who were assisted during most of this time by a special staff of four physicians, three nurses, three social workers and two accountants. It is quite as interesting and almost as valuable to the Briton as to the citizen of the United States.

### CALLING IN QUESTION.

**Equality and Fraternity.** By Douglas Macleane, Canon of Salisbury. (George Allen and Unwin. 7s. 6d. net.)

Canon Macleane is a vigorous and challenging writer who should set many of us asking what is the value of the ethical shibboleths by which we set so much store. He attacks many of them boldly from a point of view higher and more sublimated than that of mere ethics. He discusses the rights of man, equality, fraternity, the aristocracy of worth, plutocracy, equal nationhood and many cognate problems with conviction and lucidity and a considerable volume of literary illustration. We cannot attempt to follow him through the whole of this wide field, but to anyone in need of refreshing and bracing reading we would commend those chapters upon various aspects of equality which occupy a large proportion of his book. He points out, what others have said before him, but which the modern world is anxious to forget, that liberty and equality, being antithetical, can never co-exist. Political equality we have got, but social equality will never be possible so long as class distinctions "continue to be marked by faint *nuances* of verbal inflection, the most distant suspicion of a twang, a hundred other trifles which one would be ashamed to set down on paper." Moreover, there can be no community without subordination of its various parts.

Canon Macleane will have none of the modern "liberal theological" and paradoxical theory that Christ is divine because of His humanity. He even argues that equality of opportunity is less frequent than formerly, and that "for one who now struggles to the top through the complicated barriers and meshes of modern society, twenty did so when life was simpler and advertisement less necessary." But was not that because the few who obtained a liberal education almost necessarily had the world at their feet? Clearly, we cannot all be made to start level; in life's race the swift and the strong must ever win. Canon Macleane says, bluntly but truly, that some must skin rabbits and others be kings or bishops or leaders of thought. However carefully we may correct "nature by nurture," an immense range of inequalities must remain. Even the "educational ladder" too often favours mere mediocrity.

Canon Macleane is by no means the first to indict modern materialism, but he does it with a vigour and gusto which make him excellent, if disquieting, reading. He may not exactly disquiet the "New Woman," but he will at least make her exceedingly angry, which will probably be very good for her. The modern young man, he says, looks for goddesses and finds only hoydens. We hope that is true. But can we be sure that, on the whole, the young man is any more intellectual than the girl who is imperfectly conscious of being a woman, and does not much want to be courted with the old courtesy, delicacy, and respect? We may have talked a little too much in the past about what Canon Macleane calls "the mystery of sacred womanhood," but perhaps we talk too little about it now. We have only to go a little farther to reach a point of view which would regard young women equally with young men as "cannon-fodder." That would be too much for the world, but much modern talk on this subject follows lines that are almost as grotesque. Canon Macleane's readers will be divided into those who agree heartily with him and those who regard him as a pale imitation of an ineffectual antichrist, but he will probably not be much concerned. He has disturbed the waters of complacency and forcibly indicted that modern self-sufficiency which imagines that the reigning generation is always right, but that the next one will be even wiser. But wisdom sometimes lies behind as well as before, and he has done a bold and useful thing by asking us to look upon some of the shibboleths of modernity in the light of history, sane philosophy, and that human nature which is essentially changeless.

### TWO EMINENT BENEFACTORS.

**Lord Lister.** By Cuthbert Dukes, M.D., M.Sc., D.P.H. Roadmaker Series. (Leonard Parsons. 4s. 6d. net.)

**William Harvey.** By R. B. Hervey Wyatt. Roadmaker Series. (Leonard Parsons. 4s. 6d. net.)

Though Lord Lister's name is familiar, even to the man in the street, as the originator of antiseptic surgery, and though a good deal has been written about his work, there was ample room for Mr. Cuthbert Dukes's book, which follows, with no irrelevancies or unnecessary details, every phase of Lord Lister's career as student, surgeon and tireless worker in the elimination of post-operative danger from all forms of sepsis. As Mr. Dukes says in his short preface: "Lord Lister's life was not an eventful one. All his time and energy were devoted to his work, and the tale of his life is the tale of his work." It is also the tale of thirty-seven years of happy married life with the daughter of his friend, Professor Syme, then considered to be the best surgeon in the British Isles. A former teacher of Lister's said, in the mid-'seventies, that operative surgery had reached finality. There were regions in the human body into which the surgeon could never penetrate — the abdomen, the chest and the brain. Deaths in hospitals from septic causes after operations were so general that Sir James Young Simpson classified pyæmia, septicæmia, tetanus, gangrene and erysipelas under the name of hospitalism, and declared that "the man laid on an operating table in one of our surgical hospitals is exposed to more chances of death than the English soldier on the field of Waterloo." How Lister gradually overcame the obstacles in his path and finally arrived at his methods of antiseptic surgery which, combined with the aseptic methods of Von Bergmann, have made a revolution in surgery, and how he won personal popularity and honours, are here clearly shown.

Servetus, Columbus and Cæsalpinus have all been acclaimed as discoverers of the circulation of the blood. Even philosophers of so long ago as Aristotle and Plato spoke of the heart as the source as well as the reservoir of the blood and distinguished between arteries and veins, but it remained for William Harvey to discover for us the exact method of the circulation of the blood—a discovery which lifted medical and natural science from confusion and gave scientists a definite starting-point. The author of this book shows us how, like Lister, Harvey gained one important hospital appointment after another, held an honoured position at Court, and lived beyond his eightieth birthday after devoting his life to scientific work of which we are all reaping the benefits to-day. He lays particular stress on Harvey's method of work and on the fact that his discovery prepared the way for subsequent physiologists. Of the two books the one dealing with Lord Lister is, perhaps, the more attractive to the general reader, since he appears to have had a more likeable personality, and his discoveries appeal more to the unscientific mind than those of Harvey.

### THE RISE OF MAN.

**The Evolution of Man: Essays.** By G. Elliot Smith, M.D., F.R.S. (Oxford University Press. 8s. 6d. net.)

The reconstruction of the lines along which the present races of mankind have evolved is a fascinating problem. The traces are so scanty and the remains so fragmentary that they allow ample scope for theory, and also, it may be said, for imagination. The present series of essays forms, however, a carefully reasoned statement of the facts by one who has devoted many years to their consideration. Professor Elliot Smith for a long time held the professorship of anatomy at the Medical School in Cairo, where he was brought into touch with the remains of the earliest civilisations in the Nile Valley. From these it was an almost inevitable step to a consideration of the more general question of man's ancestry and of the skeletal vestigia of his forerunners.

The most important contributions which Professor Elliot Smith has made to the elucidation of the problems involved have, however, been in connection with the changes through which the vertebrate brain has passed in the course of evolution, and the supersession of the primitive Rhinencephalon by the growth of the Neopallium. He has shown that the assumption of stereoscopic vision by the primitive ancestors of the Primates, so valuable an adjunct to arboreal life, led to an increasing specialisation and greater freedom in the use of the fore limbs for fine and delicate movements, which in turn prompted further development of the brain. Each advance reacted upon the other—

> Till at the last arose the man
> Who throve and branched from clime to clime.

The fact that the essays, except the introductory chapter, are reprints of lectures delivered at various times and in various places, naturally causes a certain degree of overlapping and repetition. There is, however, a general continuity of treatment, and the book, which is well illustrated and indexed, will prove invaluable to all who take an intelligent interest in the marvellous story of man's origin and development. In this study they can have no surer guide than Professor Elliot Smith.

### SOME MODERN MONOGRAPHS.

**Modern Methods in the Diagnosis and Treatment of Pulmonary Tuberculosis.** By R. C. Wingfield, M.B., M.R.C.P. (Constable. 10s. 6d. net.)

**Modern Diagnosis and Treatment of Syphilis, Chanchroid and Gonorrhoea.** By L. W. Harrison, D.S.O., M.B., Ch.B., M.R.C.P. (Constable. 10s. 6d. net.)

**Modern Views on the Toxæmias of Pregnancy.** By O. L. V. de Wesselow, M.B., F.R.C.P., and J. M. Wyatt, M.B., F.R.C.P. (Constable. 7s. 6d. net.)

Under the able editorship of Dr. Hugh Maclean, Messrs. Constable have just published a series of "Modern Medical Monographs," the primary object of which is to provide the busy practitioner with a concise and contemporary account of the subject under consideration. Dr. Wingfield contributes the first volume upon pulmonary tuberculosis, and makes it quite clear that even in a case of well-advanced disease with positive sputum it is possible to have only slight or no physical signs. In such circumstances the X-rays prove of considerable assistance, and for this purpose plates or films are better than a screen examination. Rest is still the mainstay of treatment, and artificial pneumothorax has proved a valuable means of saving life. Altogether this is a most helpful text book.

Lieut.-Col. L. W. Harrison has been selected as the author of the monograph upon venereal disease, the three main forms of which are here succinctly described, together with the principles of treatment. The importance of the maintenance of bodily health by every means possible during arsenobenzol treatment is emphasised, as thereby immune substances are formed which are of considerable protective value. The patient's tissues have to be considered, as well as the pursuit and destruction of the spironeme. Prompt auto-disinfection after exposure may diminish the risk of infection, but the author does not believe that it has the magical effect ascribed to it by the advocates of the method. This book may be commended to the practitioner who wishes to treat his venereal patients safely and effectively.

The various morbid conditions grouped under the heading of the toxæmias of pregnancy have afforded a wide field for research as well as for speculation. In the hands of Drs. de Wesselow and Wyatt, of St. Thomas's Hospital, these abnormal states, which include hyperemesis, eclampsia, and albuminuria, are fully discussed, together with the theories that have been advanced as to their causation. The chief guide in recommending induction of labour in the pre-eclamptic state is stated to be the blood-pressure, whereas the results of chemical examination, though valuable in diagnosis, are of little prognostic importance. Stroganoff's and the Dublin methods of expectant treatment of eclampsia are given in detail. As a means of enhancing the safety of pregnant women this book should be widely read by every general practitioner.

### OTO-RHINO-LARYNGOLOGY.

**Diseases of the Nose, Throat and Ear.** Edited by A. Logan Turner, M.D., F.R.C.S.Ed. Illustrated. (John Wright and Sons, Ltd. 20s. net.)

The popularity of the small volume on diseases of the throat, nose and ear, written by the late Major W. G. Porter, D.S.O., has never been disputed, and since its third edition is out of print, the idea of producing a joint work upon this speciality, dedicated to his memory, was promulgated among those engaged in this department of the Edinburgh Medical School. Dr. A. Logan Turner in his task of editor of the present volume has been fortunate in obtaining the collaboration of five well-known lecturers and teachers, each of whom is responsible for one of its main sections. The editor and Mr. W. T. Gardiner contribute the section on diseases of the nose, and in this, as in other divisions of the book, the wise plan is adopted of first describing the normal anatomy of the parts, followed by an account of the modern methods of investigating them to detect the presence of any abnormality. The value of skiagraphy is pointed out in affections of the para-nasal sinuses, particularly in the diagnosis of suppuration in the frontal sinus. A good description is given of the condition known as " vacuum frontal headache " due to closure of the inlet to the frontal sinus. The section on the pharynx and naso-pharynx is contributed by Dr. Douglas Guthrie. Small tonsils are stated to be often the worst offenders as regards the presence of septic infection, a fact with which most clinical observers will be in accord. In dealing with the all-important question whether tonsillectomy should be performed, the author says that although some alteration of voice may take place which, in the case of trained singers, may necessitate a further period of training, the ultimate result of the operation will be good. The dissection method, it may be mentioned, is not the routine one generally adopted in Edinburgh where the guillotine finds more favour than in London.

In the section on diseases of the larynx, contributed by Mr. J. D. Lithgow, will be found some ingenious diagrams explanatory of the action of the laryngeal muscles. The difficulties commonly experienced in laryngoscopy are pointed out with their appropriate remedies. The excellent coloured plates showing various stages and forms of tuberculosis of the larynx deserve careful attention and form an indispensable supplement to the description of the disease in the text. Heliotherapy is not mentioned as a remedy for this condition, but the use of intra-tracheal injections of 10 per cent. menthol in paroleine is recommended. Mr. G. Ewart Martin contributes the section on peroral endoscopy, which includes the various methods by which a direct inspection may be made of the larynx, trachea, lower air passages and œsophagus. Blind attempts at the removal of foreign bodies in the œsophagus by means of coin-catchers and probangs are strongly condemned, but the value of direct inspection is impressed upon the student.

The section on otology has been entrusted to Dr. J. S. Fraser and consists of nine chapters, each of which bears the imprint of the experienced teacher. A great many of the so-called aids to hearing can only be described as " humbug apparatus," the instruments being all small, very costly and of little use. The functional examination of the vestibular apparatus is carefully described in considerable detail. Middle-ear suppuration and its treatment is thoroughly dealt with. Throughout the book the illustrations, both plain and coloured, are really helpful, and there is a useful list of various formulæ at the end. Altogether we have no hesitation in recommending this text-book as a masterly introduction to the study of ear, nose and throat diseases, and it will be welcomed alike by practitioners and students.

### PENAL METHODS.

**The Soul of a Criminal.** By John C. Goodwin. (Hutchinson. 18s. net.)

With the publication of this volume Mr. Goodwin completes his trilogy on criminal matters, but we fail to see that he has taken us much further than in his former book, *Insanity and the Criminal.* He enters here an earnest plea for the recognition of the newer psychological theories on the evolution of delinquency. It is a most curious medley of musings on the structure of the brain, the relations between mind and body, and the Freudian theory, and the author informs us that he takes " soul " as equivalent to " mind, nature, personality, individuality, the Ego." The book is described as " authoritative." But we cannot regard the writer as equipped for the exposition of scientific subjects. The information given is inadequate and in many instances quite inaccurate. For example, we are told that imbecility is due to deficiency of the thyroid gland, the lay reader—for whom we presume the book to be intended—being left under the impression that all cases of imbecility are due to this cause, with the implication that the condition is curable by appropriate thyroid treatment. Moreover, the term " psychosis " is used quite indifferently as a synonym for delusion, hallucination and obsession

Yet with all its faults the book is well-meaning, is written in a vivacious style, and may do some good. The author feels deeply the glaring inadequacies of our present penal methods. He realises, and urges, that the real subject of attention should be the offender and not the crime; and he quite correctly contends that we cannot even hope to deal with the offender on rational lines until we have taken the trouble to understand his mentality. Some of Mr. Goodwin's readers may be disposed to give further thought to this subject, and may be induced to pursue their reading into some of the really authoritative books which are mentioned in a well-selected bibliography.

### APPLIANCES FOR THE DISABLED.

**Artificial Limbs.** By Dr. Florent Martin. (Geneva: International Labour Office. 5s. net.)

That the fitting and construction of any artificial appliance designed to replace the whole or part of a natural limb which has been lost by disease, accident, or warfare, should be based upon a sound knowledge of its functions would appear to be a truism. Yet it must be confessed that in many parts of the world the manufacture of artificial limbs is still in the hands of mechanics some of whom have had little or no scientific training. The publication of the present volume by the International Labour Office is the outcome of a series of conferences on questions affecting the welfare of disabled ex-Service men, and the author has set forth a mass of information available for anyone who wishes to know the best form of appliance for any given mutilation.

It is obvious that the possession of a perfectly fitting artificial limb, constructed upon the latest scientific principles and of proved value to the wearer, will be of the greatest assistance in restoring the vocational capacity of disabled men, and Dr. Martin, who was attached to the limb-fitting branch of the Belgian Army Medical Corps, and is now Director of the Technical and Scientific Institute of Artificial Limb Fitting set up in Brussels in 1921 by the Permanent Inter-Allied Committee for Disabled Men, has succeeded in giving a detailed account, accompanied by excellent illustrations, of the various appliances needed for every possible condition of mutilation. Most ingenious are the mechanical hands, and the Pringle-Kirk arm, invented by two Irishmen, one a medical man and the other an engineer, is a triumph of originality.

---

### MISCELLANEOUS NOTICES.

#### The Perfect Meal.

Vitamins: What We Should Eat and Why. By R. H. A. Plimmer, D.Sc., and V. G. Plimmer. (People's League of Health. 1s.)—There should be no excuse, after the publication of this little book, for the continuance of any of the errors of diet for which we have lately been constantly reprimanded. It is the result of a resolution passed at the First International Conference of the People's League of Health at the Wembley Exhibition to the effect that "a committee be formed to prepare and produce a pamphlet on proper food as the basis of health, and the first step in the prevention of disease." Having learnt our lesson from a book by such authorities as Mr. and Mrs. Plimmer, with an introduction by Sir Arbuthnot Lane, we ought to feel quite capable of preparing, or at least ordering, meals calculated to keep us perfectly healthy. The book has, in fact, an honest title, for it tells us shortly and exactly what we should eat and why, and concludes with classified lists of foods and the particular vitamins they supply.

#### A Northern Hospital.

Colne Jubilee Cottage Hospital, 1897–1924, and Hartley Hospital, 1924. An epitome. Compiled by H. W. Croasdale. (1s. 6d.)—Hospital histories are always welcome because they are still too few, and we must congratulate the secretary of the Hartley Hospital, as it has become, upon having compiled this epitome, now that its first phase as a cottage hospital has come to an end. Originally a Memorial of the Diamond Jubilee, the institution has throughout owed much to the Hartley family. It was their policy to give, if the locality would undertake to maintain, and both sides of this arrangement have been fulfilled with great generosity. The original cottage hospital and the new Hartley Hospital were both founded on this plan. At the end of last year the endowment fund had reached £15,000, and the contributory scheme is flourishing. The epitome, which is finely illustrated, can be obtained from the hospital, and the proceeds benefit the hospital's funds. By avoiding too much detail, the principle of growth which has existed at Colne from the first is made by Mr. Croasdale into a continuous and interesting story.

#### Some Christmas Fiction.

The Dream Man. By Pamela Wynne. (Philip Allan. 7s. 6d. net.)—Silver Star Dust. By Cecil Adair. (Stanley Paul. 7s. 6d. net.)—Queen's Nurses' School. By E. Everett-Green; Murray Finds a Chum. By May Wynne; Nipper and Co. By May Wynne; The Heroine of Chelton School. By May Wynne.; Crystal's Victory. By Cecil Adair. (Stanley Paul. 2s. net each.)—Miss Pamela Wynne tells a story with real spirit. That her story is utterly unlike life may the more commend it to the general public, who may even credit the amazing coincidence by which Monica and her "Dream Man"—actually a bad-tempered and difficult creature—are restored to each other. There is. however, a certain air of reality about Monica, foolish and irritating as she is, which suggests that Miss Wynne has an insight into character and makes her book entertaining to those who are unimpressed by the intricacies of her plot. "Silver Star Dust" is another of Miss Adair's blameless romances, sure to find favour with her very numerous readers. The other stories, the titles of which appear at the head of this notice, foim part of "The Endsleigh Rewards," an excellent series of tales for children, which is being issued by Messrs. Stanley Paul. These are by well-known authors, attractively bound and clearly printed, while their price is exceedingly moderate.

#### "Problem" Cases.

Case Studies. The Judge Baker Foundation, Boston, U.S.A. $2.50—Drs. William Healy and Augusta Bronner present us with a most admirable set of twenty case studies, selected from the work which they are doing at Boston. The Judge Baker Foundation exists for the study of "problem" cases occurring among adolescents. The cases with which it deals do not come exclusively from the courts, and many of them are not connected with legal wrongdoing. It is found that parents, teachers and others are quite ready to take advantage of the facilities for expert advice which have been provided for them. The studies are of the most detailed character, covering every aspect of the patient's history and condition. No reader can fail to be impressed by the careful work which they describe. By an ingenious arrangement the facts obtained by clinical study of the case are presented separately from the conclusions which have been drawn therefrom. Thus the reader can form his own conclusions from the facts and can then see how far they agree with those of the authors. The studies are of the utmost interest to all students of abnormal conduct. We may express the hope that they will be studied by magistrates, probation officers and others who are concerned with the treatment of adolescent offenders. The study of the individual is the only way in which the problems presented by misconduct can ever be solved. What is being done in America could be done here—it is only a question of expense. The expenditure required would not only be small, but would be remunerative. By adopting American methods we should ultimately save a large sum which is now spent in futile methods of attempting to deal with these problem cases. We learn slowly, and it can only be hoped that, when we do move, we shall do so surely and with conviction.

